# Yoga therapy to reduce fatigue in cancer: effects of reminder e-mails and long-term efficacy

**DOI:** 10.1007/s00520-021-06345-z

**Published:** 2021-06-22

**Authors:** Teresa Zetzl, Andre Pittig, Agnes Renner, Birgitt van Oorschot, Elisabeth Jentschke

**Affiliations:** 1 Interdisciplinary Center, Palliative Medicine, University Hospital Würzburg, Josef-Schneider-Str. 11, B1, 97080 Würzburg, Germany; 2 Department of Psychology (Biological Psychology, Clinical Psychology, and Psychotherapy), University of Würzburg, Würzburg, Germany; 3 Center of Mental Health, University of Würzburg, Würzburg, Germany; 4 Comprehensive Cancer Center Mainfranken, University Hospital Würzburg, Würzburg, Germany

**Keywords:** Mind–body intervention, Complementary alternative medicine, Long-term effects, Yoga, Fatigue, Reminder e-mails

## Abstract

**Objective:**

To examine the efficacy of reminder e-mails to continue yoga therapy on practice frequency and fatigue in cancer patients and long-term effects of yoga on fatigue, depression, and quality of life.

**Methodology:**

One hundred two cancer patients who completed an 8-week yoga therapy were randomly allocated to two groups: reminder (*N* = 51) vs. no-reminder group (*N* = 51). After completing yoga therapy, the reminder group received weekly e-mails for 24 weeks, which reminded them of practicing yoga, whereas the no-reminder group did not. Primary outcomes were fatigue and practice frequency, and long-term outcomes were fatigue, depression, and quality of life. Data were assessed using questionnaires after yoga therapy (T1) and 6 months after completing yoga therapy (T2).

**Result:**

A significantly stronger reduction of general (*p* = 0.038, *d* = 0.42) and emotional fatigue (*p* = 0.004, *d* = 0.59) and a higher increase of practice frequency (*p* = 0.015, *d* = 0.52) between T1 and T2 were found for the reminder group compared to the no-reminder group. In the mediation model, practice frequency as a mediator partially explained the changes in emotional fatigue (indirect effect *B* =  − 0.10). Long-term effects of yoga therapy regarding fatigue, depression, and quality of life were found (*F* > 7.46, *p* < 0.001, *d* > 0.54).

**Conclusion:**

Weekly reminder e-mails after yoga therapy can positively affect general and emotional fatigue and help cancer patients with fatigue establish a regular yoga practice at home. However, higher practice frequency did not lead to higher physical or cognitive fatigue improvement, suggesting other factors that mediate efficacy on physical or cognitive fatigue, such as mindfulness or side effects of therapy.

## Introduction

Detection and treatment of cancer have significantly improved, leading to increased survival time in cancer patients. However, contemporary treatment methods are not without side effects [1]. As a result, side effects of cancer and their treatment gain more and more attention. Fatigue, one of the most common side effects of cancer and cancer-related treatment, is described as intense and chronic tiredness on a physical, emotional, and cognitive level, which is not related to previous activities and cannot be entirely reduced by sleep [2]. Twenty to eighty percent of cancer patients suffer from fatigue during therapy [3], and fatigue can persist even 5 years after therapy completion [4]. Increased interleukins [2, 3], anemia, and psychological stress [4, 5] might play a significant causal role in the etiology of fatigue, but the complexity of this interaction is not yet fully understood. Therefore, symptom-oriented treatment of fatigue is usually preferred to cause-specific treatment. The National Comprehensive Care Network recommends yoga as a category 1/grade A non-pharmacologic intervention for cancer patients during and after cancer-related therapy. Yoga interventions of different styles and duration were effective in reducing fatigue in cancer patients [6–10]. Meta-analyses of yoga with cancer patients report small to moderate effects on fatigue [11–14].

Findings on the long-term effects of yoga in cancer patients are less coherent than those for short-time effects. Some randomized controlled trials report a significant reduction in fatigue after yoga therapy, but no effect after 3 months compared to control groups without interventions [15, 16]. Another randomized controlled trial finds no significant improvement in fatigue directly after yoga compared to a control group, but 3 months later [17]. In an observational study without a control group and with pre-post measurements, yoga intervention also showed significant long-term effects on fatigue and on depression, and anxiety months after completing of the yoga intervention [18].

The sustainability of such positive effects of yoga interventions is related to patients’ practice frequency during and after the intervention [17, 19]. Patients suffering from fatigue are physically, emotionally, and cognitively exhausted. Therefore, it is quite comprehensible that patients affected by fatigue have difficulties motivating themselves to practice independently outside of regular classes [20]. Knowledge about physical activity and its impact on quality of life, physical activity before diagnosis [20], social support, and a given structure and appointed times for sports classes [21] have a positive influence on the patients’ motivation for physical activity. Clinical depression, in contrast, keeps patients from physical activity [20]. To facilitate patients’ independent practice, it is an established method to provide them with an exercise CD and exercise book at the end of a yoga intervention [16, 22–24]. Individual documentation of the daily exercise duration in addition to the exercise CD was also proven to be helpful concerning the sustainability of positive effects [17]. Although these methods aim to establish regular yoga exercises, they are usually offered only once at the end of the yoga therapy, and due to a lack of regularity and frequency, they do not represent a reminder of the yoga practice.

Daily reminder e-mails help increase adherence to medication [25, 26]. Cicolini et al. (2014) also used weekly reminder e-mails to remind patients with cardiovascular risk factors to adopt a healthy lifestyle. This led to significantly lower cardiovascular risk factors compared to a group that did not receive reminder e-mails. However, there are no comparable studies on yoga for improving fatigue symptoms that use weekly reminder e-mails to promote lasting effects.

In a previous paper, we described the efficacy of yoga therapy regarding fatigue, depression, and quality of life. Patients who participated in an 8-week yoga therapy reported significantly lower general and physical fatigue and depression and higher quality of life compared to patients in a waiting list control group. A higher attendance rate was associated with more reduction in fatigue [10]. This paper builds on the results of the previous paper and examines the stabilization of the efficacy of yoga therapy through reminder e-mails as described above and the long-term effects of yoga therapy as an additional research question.

Thus, the first research question of this study should examine the efficacy of reminder e-mails on fatigue and its subscores compared to patients who do not receive reminder e-mails [27]. We hypothesized that patients who receive reminder e-mails would report lower fatigue scores. Our second research question aimed to examine the efficacy of reminder e-mails on increasing practice frequency compared to patients without reminder e-mails. The hypothesis was that patients with reminder e-mails practice more frequently than patients without reminder e-mails. The third hypothesis was that the effect of reminder e-mails on fatigue and its subscores would be mediated by practice frequency. The fourth research question addressed the long-term changes in self-reported fatigue, depression, and quality of life after an 8-week yoga intervention for patients with different types of cancer after 6 months. We hypothesized that fatigue would be significantly lower immediately after yoga therapy and at the follow-up 6 months later compared to the baseline.

## Methods

### Design and procedure

This randomized controlled trial took part in the University Hospital Würzburg from November 2018 to September 2020. After informed consent, patients participated in 8-week yoga classes for 1 h a week. At the end of the yoga therapy, participants received a booklet and a CD with instructions of the asanas they learned in class. Afterward, participants were randomly assigned to “reminder” or “no-reminder” group. A block randomization procedure was used. Patients assigned to a yoga group form a block. The randomization list with computer-generated numbers was compiled by staff members of the Palliative Medicine Centre. The reminder group received a weekly reminder e-mail over 24 weeks. Primary (fatigue, practice frequency) and secondary outcomes (depression, quality of life) were assessed before and after yoga therapy and 24 weeks after the end of yoga therapy via self-report questionnaires. The Ethics Commission of the University of Würzburg has approved the study in advance (59/18-sc). The study design can be found in the protocol article [28].

### Participants

Participants of this randomized controlled trial were adult cancer patients, at least 18 years old, who were planning to undergo cancer treatment in University Hospital Würzburg at the time of screening. In order to be included, patients had to report at least mild fatigue symptoms in the Fischer screening (intensity ≥ 1, impairment ≥ 1) [29]. Exclusion criteria were insufficient knowledge of the German language, severe emotional or physical impairment, and more than 50-km distance to the university hospital.

### Yoga intervention

The yoga intervention ran for 8 weeks. Patients took part in one session of 1 h per week. The yoga intervention consisted of different asanas (physical postures with awareness), small series of conscious breathing (pranayama), and deep relaxation (savasana) at the beginning and the end of each class, inspired by a mindfulness-based stress reduction program (MBSR) [30]. The exercises were structured from lying to sitting to standing and kept constant for all sessions. In all exercises, participants were reminded to breathe slowly, deeply, and consciously. Nonviolence (ahimsa), as an important basic principle of yoga, was repeated in every session and helped to encourage the participants to be gentle with their bodies and personal limitations.

### E-mail reminder

After completing of yoga therapy, personal e-mails were sent on the same day every week, for 24 weeks altogether. In the first 12 weeks, e-mails contained descriptions of twelve yoga exercises—one exercise each week—and a personal encouragement to practice yoga independently during this week. The yoga exercises, known to the participants from the yoga classes, were described analogously to the description in the group yoga classes. In the following 12 weeks, the twelve reminder e-mails with these exercises were repeated in the same order.

### Questionnaires

Outcomes were assessed directly before and after yoga therapy (T0 and T1) and 6 months later (T2).

#### Primary outcomes

##### Self-reported fatigue

Self-reported fatigue was assessed using the German version of EORTC QLQ-FA13—13 items (European Organization for Research and Treatment of Cancer–Quality of Life Questionnaire–Fatigue) [31]. This questionnaire can be used in all tumor diseases in all stages and phases of the disease and all areas of treatment (chemotherapy, radiation, surgery) or care (acute care, rehabilitation, aftercare, or palliative care) [32]. The EORTC QLQ-FA13 measures general fatigue using 13 items overall. Physical fatigue was assessed with five items (e.g., have you felt exhausted?), emotional fatigue with three items (e.g., did you feel discouraged?), and cognitive fatigue with two items (e.g., did you have trouble thinking clearly?). Response categories of all items are “not at all,” “a little,” “quite a bit,” and “very much,” coded with scores from 1 to 4. Mean scores are calculated for each subscale and the overall scale and then linearly transformed to symptom scales ranging from 0 to 100. Higher values indicate a higher level of fatigue symptoms. The internal consistency for the German version was good, with Cronbach’s alpha values ranging from 0.79 to 0.90 [31].

##### Practice frequency

The practice frequency was assessed after yoga therapy and 6 months later with open responses. Patients indicated how many days they had practiced in an average week and how many minutes they practiced on average per practice day. The variable was calculated by multiplication of average days and average minutes.

#### Secondary outcomes

##### Depression and quality of life

Depression was assessed using the Patient Health Questionnaire (PHQ-9), which assesses symptoms of depression according to the DSM-V [33]. Higher values indicate higher depression. The internal consistency for the PHQ-9 proved to be good with Cronbach’s α = 0.79 for cancer patients [34].

Quality of life was assessed with the function scale of EORTC QLQ-C15-PAL [35]. This scale consists of the item *How would you rate your overall quality of life during the past week*?, scaled from 1 (very poor) to 7 (excellent). Higher scores represent a higher quality of life.

##### Sociodemographic and health data

The following sociodemographic data was assessed at T0: age, gender, marital status, number of children, educational level, professional status, tumor diagnosis. In addition, personal benefits of yoga and reasons not to practice (anymore) were assessed at T2 on a four-point Likert scale (does not apply at all, applies completely).

### Statistical analyses

All analyses were performed on intention-to-treat basis on a significance level of α = .0.05. Data analysis was carried out using IBM SPSS Statistics version 26. For the first hypothesis, analyses of variance (ANOVA) for fatigue and each subscale (physical, emotional, and cognitive) using time (T1 vs. T2) as within-factor and type of reminder (reminder vs. no-reminder) as a between-subject factor were performed. For the second hypothesis, we calculated a *t* test for independent samples (reminder vs. no-reminder) with residual gain scores (RGS) of practice frequency [36] as the outcome variable. RGS were calculated by subtracting the standardized *z* values of the practice frequency at T1 multiplied with the correlation of T1 and T2 from the standardized *z* values of the practice frequency at T2. RGS control baseline differences and measurement errors occurring in the use of repeated measures on the same instrument [36]. The third research question was tested using a mediation model with group (reminder vs. no-reminder) as a predictor, RGS of practice frequency as a mediator, and RGS of fatigue and subscores as dependent variable. Mediation analyses were performed using the PROCESS macro by Hayes (2018) [37]. The 95% confidence interval of the indirect effects was obtained with 5000 bootstrap resamples [38]. The fourth research question was tested with an ANOVA and paired *t* tests with adjusted α*=*0.016 to analyze long-term changes in fatigue, depression, and quality of life between baseline, after yoga, and follow-up.

## Results

### Participant flow and assignment

**Fig. 1:**
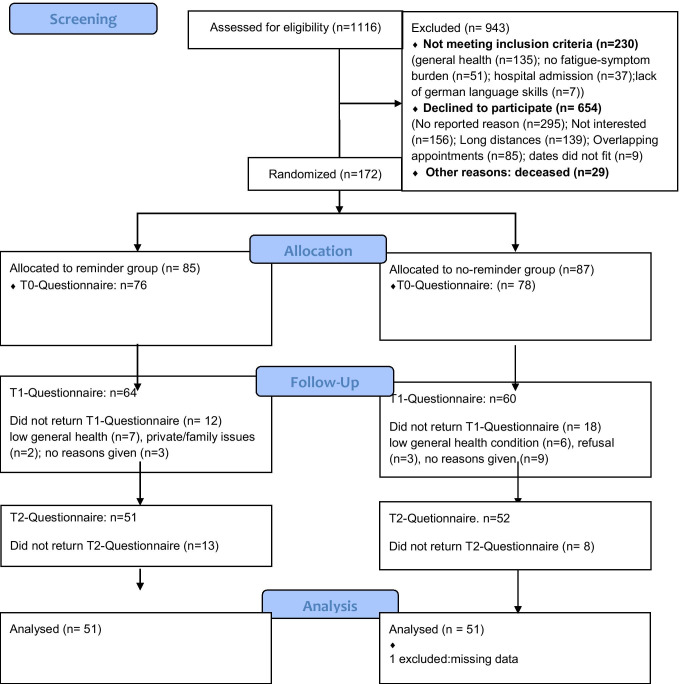
CONSORT diagram showing screening, allocation, and participant flow by group

Patients were recruited between November 2018 and December 2019. One hundred seventy-two patients agreed to participate and were randomly assigned to a reminder (*n* = 85) or no-reminder group (*n* = 87). Due to dropouts and non-returned questionnaires, a total of 102 patients (reminder *n* = 51, no-reminder *n* = 51) were finally included in the analyses. For a detailed description of participant flow, see Fig. 1.

### Demographics and clinical characteristics

Participants’ age ranged from 24 to 82 (*M* = 59.2, SD = 11.5), 72.5% were female, and 68.8% were married or in a long-term relationship. The participants were predominantly diagnosed with breast cancer (52.5%), followed by prostate cancer (13.1%) and lymphoma (9.1%). Eighty-six percent were under cancer-specific treatment at T0 (see Table 1). There were no significant differences between reminder and no-reminder groups in demographic (age, gender, marital status) or health-related (cancer, treatment state) characteristics.

**Table 1:** Demographic and clinical characteristics of study population by group

Characteristics	All (*N* = 102)	Reminder (*N* = 51)	No-reminder (*N* = 51)
Age [mean (SD)]	59.2 (11.5)	56.6 (10.7)	61.7 (11.7)
Range	24–82	34–81	36–82
	% (*N*)	% (*N*)	% (*N*)
Female	72.5 (74)	78.4 (40)	66.7 (34)
Marital status			
Married/partnered	68.8 (70)	70.6 (36)	66.7 (34)
Never married/single	11.8 (12)	7.8 (4)	15.7 (8)
Divorced/separated	12.7 (13)	17.6 (9)	7.8 (4)
Widowed	4.9 (5)	2.0 (1)	7.8 (4)
Education level			
Primary education	29.4 (30)	25.5 (13)	33.3 (17)
Secondary education	27.5 (28)	25.5 (13)	29.4 (15)
Tertiary education	40.2 (41)	47.0 (24)	33.3 (17)
Others	1.0 (1)	0.0 (0)	2.0 (1)
Tumor diagnosis			
Breast cancer	52.5 (52)	60.4 (29)	45.1 (23)
Prostate cancer	13.1 (13)	10.4 (5)	15.7 (8)
Gastrointestinal cancer	7.1 (7)	10.4 (5)	3.9 (2)
Lung cancer	5.1 (5)	0.0 (0)	9.8 (5)
Lymphoma	9.1 (9)	8.3 (4)	9.8 (5)
Gynecological cancer	6.1 (6)	6.3 (3)	5.9 (3)
Head and neck cancer	3.0 (3)	0.0 (0)	5.9 (3)
Cancer of CNS	2.0 (2)	2.0 (1)	2.0 (1)
Other cancer	2.0 (2)	2.0 (1)	2.0 (1)
Metastases			
Present	18.6 (19)	15.7 (8)	21.6 (11)
Therapy status 6 months after yoga therapy			
Had a cancer-related therapy	44.1 (45)	58.8 (30)	29.4 (15)
Chemotherapy	5.9 (6)	5.9 (3)	5.9 (3)
Radiation therapy	1.0 (1)	2.0 (1)	0.0 (0)
Hormone therapy	28.4 (29)	37.3 (19)	19.6 (10)
Antibody therapy	7.8 (8)	5.9 (3)	9.8 (5)
Other	10.8 (11)	17.6 (9)	3.9 (2)

*SD* standard deviation, *CNS* central nervous system

### Practice frequency and evaluation of reminder e-mails

Of the reminder group and no-reminder group, 74.5% and 62.0% reported 6 months after therapy that they were currently practicing yoga. Patients in the reminder group reported an average practice of 39.7 min per week at T1 (SD = 44.7) and 68.5 min (SD = 57.0) at T2. In the no-reminder group, the self-reported average practice time was 36.3 min at T1 (SD = 43.1) and 39.9 min (SD = 39.1) at T2. The main reason for not practicing yoga anymore in both groups was lacking motivation without professional instruction (reminder: 55.6%, no-reminder: 58.6%). For more perceived benefits of yoga and reasons not to practice (anymore), see Table 2.

**Table 2:** Practice frequency, subjective benefits of yoga, and reasons not to practice (higher values represent higher agreement range 1–4)

	Reminder group	No-reminder group
Yoga practice frequency	*N* = 51	*N* = 51
Practiced yoga in the last 6 months (%(N))	88.2 (45)	72.5 (37)
Practiced yoga currently at the moment (%(N))	74.5 (38)	62.0 (31)
Average amount of yoga practice a week in minutes (m (SD))	68.5 (56.9)	39.9 (39.0)
Subjective benefits of yoga (m(SD))	*N* = 47	*N* = 44
Better concentration	2.72 (0.90)	2.57 (0.87)
Less tired	2.77 (0.84)	2.65 (0.84)
Feeling more fit	3.47 (0.78)	3.27 (0.79)
Better dealing with cancer disease	2.98 (0.87)	3.02 (0.92)
Better dealing with anxiety concerning my cancer disease	2.94 (0.90)	2.73 (0.90)
Less worried about my future	2.89 (0.91)	2.81 (0.88)
Less sad/depressed	3.11 (0.84)	3.05 (0.82)
Reasons to stop practicing yoga (m (SD))	*N* = 18	*N* = 29
Not motivated without professional instruction	2.11 (1.4)	2.66 (1.0)
Physically not able to do yoga	1.68 (1.25)	1.90 (1.0)
No need for yoga due to good state of health	1.24 (0.75)	1.82 (0.77)
No benefit of yoga therapy	1.44 (1.11)	1.30 (0.61)
Subjective experienced harm through yoga	1.12 (0.93)	1.21 (0.49)
No time for yoga in everyday life	2.06 (1.56)	1.89 (0.89)

*SD* standard deviation, *NA* not applicable

In the reminder group, 74.0% reported having read the e-mails always or frequently, only 8.0% indicated they never or almost never read them, 67.3% reported that they were strongly or very strongly reminded of yoga therapy by the e-mails, and 54% found the e-mails (very) helpful to keep up their independent yoga practice. Eighty-six percent of the patients felt that the e-mails were not at all or very little annoying.

### Primary outcome: efficacy of reminder e-mails on fatigue and yoga practice

#### Fatigue

**Fig. 2:**
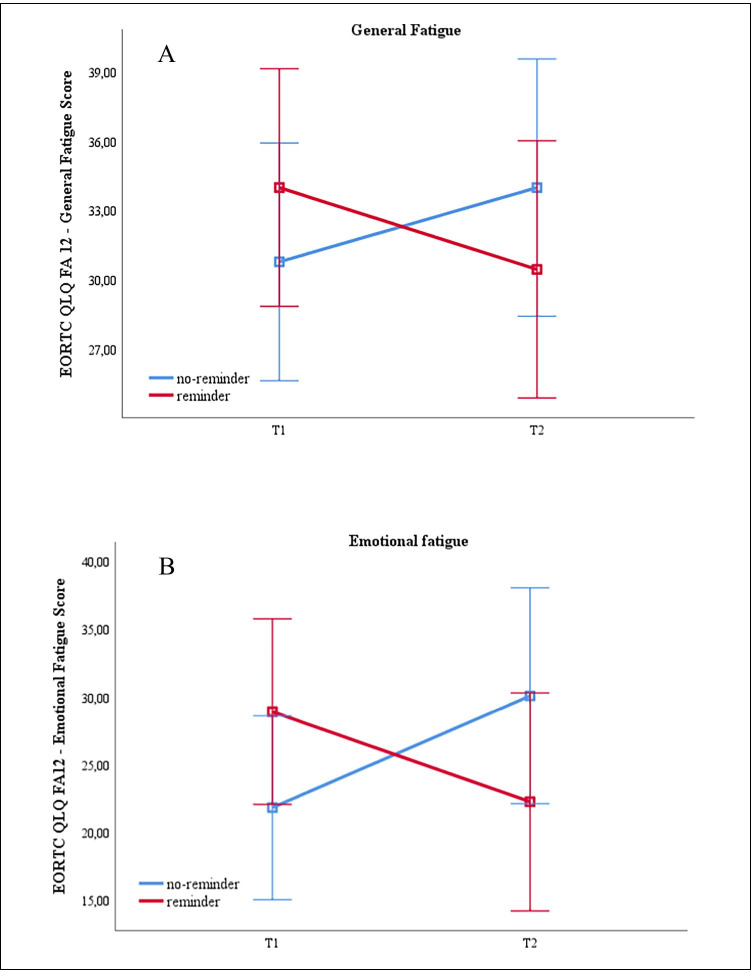
Mean changes in **A** EORTC QLQ-FA12 general fatigue and **B** EORTC QLQ-FA12 emotional fatigue between T1 and T2 in e-mail group and no-e-mail group. Results show means and 95% CI

The reminder group reported a significant reduction from T1 to T2 compared to the no-reminder group in general fatigue (*F*(100;1) = 4.420; *p* = 0.038; *d* = 0.42) as well as in emotional fatigue (*F*(99;1) = 8.538; *p* = 0.004; *d* = 0.59). No significant effects were found for physical and cognitive fatigue (see Table 3/Fig. 2). Controlling for baseline differences with analysis of covariance (ANCOVA), group (reminder vs. no-reminder) differed significantly in emotional fatigue (*F*(98;1) = 6.43; *p* = 0.013; *d* = 0.51), but not in general fatigue (*F*(99;1) = 3.611; *p* = 0.06).

**Table 3:** Means (m), standard deviation (SD), and *p* values of ANOVA analyses of time and group effects, and time × group interaction between reminder and no-reminder group for fatigue and subscales

	Reminder		No-reminder		Time	Group	Time × group
	T1 m (SD) *N* = 51	T2 m (SD) *N* = 51	T1 m (SD) *N* = 51	T2 m (SD) *N* = 51	*p* value	*p* value	*p* value
Fatigue	33.97 (20.7)	30.45 (21.49)	30.78 (16.02)	33.98 (18.47)	0.919	0.962	0.038*
Physical fatigue	45.23 (24.30)	42.48 (25.12)	42.80 (20.21)	44.40 (20.98)	0.779	0.825	0.289
Emotional fatigue	28.89 (26.47)	22.22 (29.09)	21.78 (22.32)	30.06 (28.40)	0.753	0.937	0.004*
Cognitive fatigue	18.62 (21.25)	16.01 (16.65)	20.26 (13.46)	20.92 (20.51)	0.610	0.288	0.396

#### Practice frequency

The mean of RGS of practice frequency score differed significantly between the reminder group (*m* = 0.28, SD = 1.14) and the no-reminder group (*m* =  − 0.24, SD = 0.79) (*t*(86) = 2.47, *p* = 0.015, 95% CI [− 0.94; − 0.10]) with a medium effect size *d* = 0.52.

#### Influence of practice frequency on fatigue

A mediation analysis was conducted to test whether practice frequency mediates the effect of reminder e-mails on fatigue and its subscales [37–40]. Significant correlations between reminder e-mails, practice frequency, and fatigue could only be found for emotional fatigue (*r* [0.28; 0.51]; *p* < 0.02), so the mediation model was tested only for emotional fatigue. First, reminder e-mails were positively associated with RGS of emotional fatigue (*B* =  − 0.42, 95% CI [− 0.79; − 0.09], *p* = 0.013). Second, there was a significant relation between reminder e-mails and the mediator RGS of practice frequency (*B* = 0.52, 95% CI [0.10; 0.94], *p* = 0.015). In a third step, the relation between practice frequency and emotional fatigue was not significant (*B* =  − 0.18, 95% CI [− 0.36; 0.01], *p* > 0.05). The direct effect of e-mail on emotional fatigue mediated by practice frequency was lower (*B* =  − 0.32, 95% CI [− 0.69; 0.05], *p* = 0.09) than the total effect (*B* =  − 0.42). The indirect effect of e-mail on practice frequency and practice frequency on emotional fatigue was *B* =  − 0.09, 95% CI [− 0.22; − 0.004].

### Secondary outcome: long-term effects

The assumption of sphericity was not met. Therefore, all following ANOVAs were adjusted according to Greenhouse–Geisser. Regarding general, as well as physical, emotional, and cognitive fatigue, there were significant time effects (*F* > 7.46, *p* < 0.001, *d* > 0.54). In the subsequent paired *t* tests with alpha adjustment, significant effects between T0 and T1 (*T* > 3.11, *p* < 0.002) were found for fatigue and its subscores. This significant difference was also evident between baseline (T0) and 6 months after completion of yoga therapy (T2) (*T* > 3.10, *p* < 0.002). Similar significant results were found for depression (*F*(202; 1.80) = 24.95; *p* < 0.001) and quality of life (*F*(186; 1.86) = 15.58, *p* < 0.001). Regarding the paired *t* test, there were also significant differences between T0 and T1 (*T* > 4.76, *p* < 0.001) and T0 and T2 (*T* > 4.48, *p* < 0.001). Between T1 and T2, no significant differences were found on any of the scales (*p* > 0.35).

## Discussion

This randomized controlled trial examined the efficacy of reminder e-mails on yoga practice and fatigue in oncological patients with different types of cancer. Yoga for reducing fatigue is well-evaluated in cancer patients, but findings on the long-term effects of yoga are rare [17, 18, 41, 42]. Even fewer studies considered practice frequency as a potential mediator [17].

**Fig. 3:**
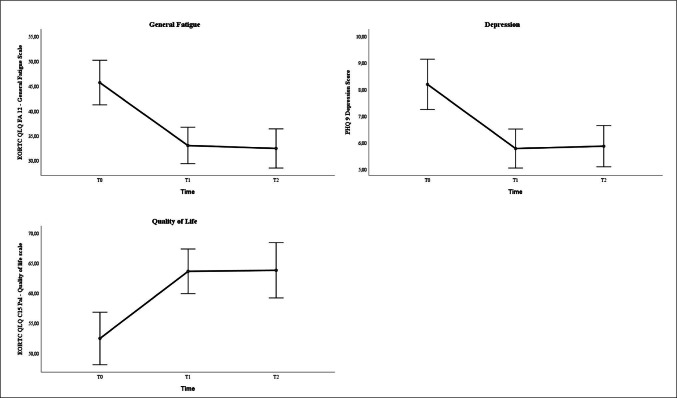
Mean changes in **A** EORTC QLQ-FA12 general fatigue, **B** PHQ-9 Depression Score, and **C** EORTC QLQ-C15-PAL quality of life between T0, T1, and T2 in all participants. Results show means and 95% CI

To the best of our knowledge, there has not yet been any study that actively tried to enhance the efficacy of yoga therapy by sending reminder e-mails. Reminder e-mails have been used predominantly in the medical context to improve compliance and adherence to appointments [25, 26, 43], but not yet regarding such a complex construct with physical, emotional, and cognitive aspects as fatigue. We found that patients who received reminder e-mails had significantly lower general and emotional fatigue scores 6 months after the end of yoga therapy with small to medium effect sizes (*d* = 0.42–0.59) compared to the group who did not receive reminder e-mails. However, we did not find significant effects regarding physical and cognitive fatigue. Furthermore, the reminder group reported a significantly higher increase in practice frequency than the no-reminder group. The mediation analysis also showed a significant relation between reminder e-mails and an increase in practice frequency. The reduction in emotional fatigue observed in this study due to reminder e-mails was partially mediated by practice frequency. Practice frequency at home may explain, at least in part, the association of reminder e-mails and emotional fatigue. However, there are other factors like mindfulness or therapy status that elucidate further variance (Fig. 3).

In several studies, higher attendance rates in yoga classes or higher practice frequency during and after yoga therapy significantly reduced fatigue [10, 17, 44]. In the current study, this could be confirmed only for emotional fatigue and general fatigue. Although reminder e-mails lead to a significantly higher practice frequency and patients in the reminder group reported lower general and emotional fatigue, a higher practice frequency was not associated with lower physical fatigue symptoms, as it was in other studies [10, 17]. Patients in the reminder group received weekly specific yoga instructions. Therefore, they may have felt more supported and less alone by the personal e-mails than the group without reminder e-mails. This may, for example, have increased their self-efficacy [44] and motivation to do yoga, which might reflect in the increased practice frequency. By actively working to improve symptoms while receiving support through reminder e-mails, they might have felt less discouraged, helpless, and frustrated than the no-reminder group, leading to an improvement of emotional fatigue.

The independent yoga exercise at home was only recorded quantitatively but not qualitatively. For successful yoga practice, mindful practice of the exercises is an important basis [30]. In the study classes, the focus was set on mindful practice through the yoga teacher’s instructions, and a higher attendance rate led to more improvement in fatigue [10]. However, this level of mindfulness in yoga practice cannot be assumed during the practice at home due to a potentially higher prevalence of disturbance factors and patients’ comparatively little expertise in mindfulness. A lack of mindfulness in performing the exercises at home could be why there was no improvement in physical fatigue. Further research is needed to test the influence of mindfulness on the efficacy of home-based yoga. In addition, it must also be critically mentioned that the exercise time during the last 6 months was asked retrospectively. This might have led to a strong retrospective bias or social desirability issues. The tendency to answer in a socially desirable way might be higher for those who received personal e-mails for 24 weeks. Thus, future research may follow up on the present results by repeatedly assessing the quality and quantity of yoga exercises during the follow-up period instead of only once at the end.

Although yoga is helpful in the beginning to reduce fatigue, it does not result in complete remission of fatigue symptoms. This is also reflected in the long-term results of this study. We found long-term improvements in fatigue, depression, and quality of life for all patients compared to baseline. Yoga can help reducing fatigue and depression and improving quality of life. These positive effects remained 6 months after the end of yoga therapy. However, there was no significant improvement between T1 and T2 after the end of yoga therapy. Yoga is helpful, especially during active cancer treatment [45], to a limited extent to reduce fatigue. Of the study population, 44.4% were still in therapy 6 months after yoga therapy. Only 40% of the participants reported a stable state of health at T2. Therefore, it is reasonable that both health status and continued therapy have a strong influence on the fatigue symptoms and that yoga can prevent further deterioration of the symptoms.

However, it must be critically considered that we did not include a control group that did not participate in yoga classes. Thus, the long-term improvement might not be solely due to yoga. Other factors, such as spontaneous remission, tumor treatment, and disease, alternative supportive offers, or social support, may have contributed to the improvement. Since most yoga studies observe brief follow-up periods from 6 to 12 weeks [16, 41, 42], we decided to use a comparatively long follow-up period of 6 months. As a result, it would not have been ethically justifiable to place these patients on a 6-month waiting list because of cancer patients’ short life expectancy. In addition to ethical reasons, the risk of low compliance with a very long waiting period also spoke against a waiting control group. Despite a high dropout between T0 and T2, of a total of 40% of the patients who agreed to the study initially, the response rate for the long follow-up of 6 months was very high at 83% compared to other studies.

In summary, previous studies supported that yoga helps reduce fatigue and that more participation in yoga leads to a higher reduction of symptoms [10]. However, our results suggest that more frequent practice does not lead to further reduction of fatigue after the end of yoga therapy but might help prevent fatigue symptoms from increasing again despite active cancer treatment. Furthermore, we have found positive effects of reminder e-mails on general and emotional fatigue after yoga therapy compared to a group without reminder e-mails. Reminder e-mails are feasible, very well accepted, and positively evaluated by cancer patients. In the long term, yoga can also help reduce fatigue and depression and improve the quality of life. Home-based yoga therapy with weekly reminder e-mails and explicit instructions for a mindful practice could be a combination that reduces the barrier of long journeys to join a yoga class in a yoga studio and increases personal motivation to individual practice.

## Abbreviations

CI: Confidence interval
EORTC QLQ-FA13: European Organization for Research and Treatment of Cancer–Quality of Life Questionnaire–Fatigue
MBSR: Mindfulness-based stress reduction
PHQ-9: Patient Health Questionnaire
QoL: Quality of life
RGS: Residual gain score
